# Characteristics of adverse reactions of three anti-glioma drugs in WHO-VigiAccess

**DOI:** 10.3389/fphar.2024.1485067

**Published:** 2024-10-24

**Authors:** Huadong Ke, Zicheng Zhang, Zhao Yu, Baiquan Zhang, Rui Chen, Qiang Zhou, Qian Guo, Xiaohui Lou

**Affiliations:** ^1^ Department of Neurosurgery, The Third Affiliated Hospital of Wenzhou Medical University, Wenzhou, China; ^2^ Nanjing University of Chinese Medicine, Nanjing, China; ^3^ Department of Neurosurgery, The First Affiliated Hospital of Harbin Medical University, Harbin, China; ^4^ Department of Otorhinolaryngology, The First Affiliated Hospital of Zhengzhou University, Zhengzhou, China; ^5^ Department of Otorhinolaryngology, The Third Affiliated Hospital of Wenzhou Medical University, Wenzhou, China

**Keywords:** glioma, adverse drug reactions, anti-glioma drugs, WHO-VigiAccess, comparative observational study

## Abstract

**Introduction:**

Antitumor medications such as Avastin, Berubicin, and Temozolomide have fundamentally transformed the treatment landscape for gliomas by exhibiting potent pharmacological effects on both high-grade and low-grade gliomas. This study aims to determine which anti-glioma medication presents the lowest risk for personalized use in clinical patients by assessing the adverse drug reactions (ADRs) associated with these medications as reported in the World Health Organization (WHO) VigiAcess database, and by comparing the characteristics of adverse responses among the three drugs.

**Methods:**

This investigation employs a retrospective descriptive analysis method. We compiled ADR reports for three commercially available anti-glioma medications from WHO-VigiAccess, gathering data on the disease systems and symptoms associated with ADRs, as well as the age, gender, and geographic characteristics of the patients represented in the reports. To provide a reference for clinical treatment, we analyzed the similarities and differences in the adverse reactions of the three medications by calculating the proportion of adverse reactions recorded for each drug.

**Results:**

A total of 132,471 adverse events (AEs) associated with three anti-glioma drugs were reported in VigiAccess. The analysis revealed that the ten most frequently reported AEs included bone marrow suppression, myalgia, leukopenia, thrombocytopenia, nausea, vomiting, death, rhabdomyolysis, disease progression, and a decrease in neutrophil count. The five most common categories of AEs related to anti-glioma drugs were blood and lymphatic system diseases (20,233 cases, 15.2%), general disorders and administration site conditions (26,973 cases, 20.3%), gastrointestinal dysfunction (22,061 cases, 16.7%), necessitating further investigations (18,285 cases, 13.8%), and musculoskeletal and connective tissue diseases (30,695 cases, 23.1%). Notably, the adverse events associated with Avastin were more pronounced in the category of musculoskeletal and connective tissue diseases compared to the other two drugs. Furthermore, Berubicin exhibited a particularly high proportion of blood and lymphatic system disease AEs, reaching 45.6%, which was significantly greater than those observed for the other two drugs.

**Conclusion:**

There is limited correlation between antineoplastic medications and ADRs. Current comparative observational studies indicate that these inhibitors are associated with both common and specific adverse effects documented in the ADR reports submitted to the World Health Organization (WHO).

## Introduction

Gliomas are central nervous system tumors originating from glial cells, which are non-neural supportive cells of the brain. The 2021 edition of WHO classification of tumors of the central nervous system divides gliomas into grades 1 to 4, grades 1 and 2 are low-grade gliomas, and grades 3 and 4 are high-grade gliomas (Louis et al., 2021). There are four grades for these groups: grade I includes more common infiltrative gliomas and pilocytic astrocytomas, pleomorphic xanthoastrocytomas, and subependymal giant cell astrocytomas; grade II includes oligodendrogliomas and astrocytomas; grade III includes anaplastic oligodendrogliomas, anaplastic astrocytomas, anaplastic oligoastrocytomas, and grade IV is glioblastoma multiforme) (Chen et al., 2017), among which the incidence of glioblastoma (glioblastoma, GBM) (WHO grade IV) is the highest, accounting for 46.1%, characterized by high incidence, high recurrence rate, high mortality rate, and low spontaneous recovery rate. Gliomas are the most common form of malignant primary brain tumors, showing diffuse and infiltrative growth, and the pathogenesis is still unclear. The tumor proliferation/apoptosis theory believes that the occurrence of gliomas is due to the imbalance of cell proliferation and apoptosis. Overexpression of anti-apoptotic genes in cells leads to reduced apoptosis, malignant proliferation of cells, and the induction of glioma occurrence. Studies have shown that angiogenesis and inflammation are crucial for the occurrence, development, and invasion of gliomas (Onishi et al., 2011), and post-transcriptional and translational modification processes are closely related to the malignant growth process of gliomas. In the clinic, broad-spectrum antineoplastic drugs Avastin, Berubicin, and Temozolomide play a huge role in the treatment of gliomas and are the most widely used drugs at present. Despite this, clinical data show that chemotherapy and other multimodal methods based on the three antineoplastic drugs, although showing prospects in extending the progression-free survival (PFS) of treating GBM, there is no evidence to show that they can extend the overall survival (OS), and the prognosis is still poor. Long-term use of anti-glioma drugs may also lead to serious adverse reactions: gastrointestinal perforation/wound complications, hypertensive crisis, nephrotic syndrome, bone marrow hematopoietic function, manifested as a decrease in platelets and white blood cells; cardiotoxicity, which can lead to heart failure in severe cases; nausea, vomiting; a few patients have fever, hemorrhagic erythema, and liver function damage. Anthracycline drugs have a dose-dependent apoptotic induction effect on glioma cells, and high concentrations of drug doses can cause cell necrosis and bring great interference to normal cells. Despite the extreme rigor of pre-marketing drug trials, the data from clinical trials cannot be used to fully assess the safety of pharmaceuticals because these trials are conducted in a controlled context that differs from the real usage environment (Gagliardi et al., 2022). Safety studies based on substantial real-world data are particularly beneficial for anti-glioma medications having a lengthy market history, a big population base, and a wide range of applications. Furthermore, less research has been done to date to compare the similarities and differences in the adverse responses brought on by the three anti-glioma medications, despite the fact that the medications have been approved for use in clinical settings. The findings of our study provide more insight into the three anti-glioma medications and their enormous therapeutic potential.

Spontaneous reporting systems are a useful tool for comparing treatment regimens, learning more about the potential reasons of adverse events, and getting actual data on the safety profile of medications and vaccines, despite their inherent limitations (Hazell and Shakir, 2006). Since their introduction in the 1960s, spontaneous reporting systems have been the cornerstone of drug vigilance. The main purpose of spontaneous reporting is to detect previously unrecognized adverse reactions at an early stage. In addition, spontaneous reporting can also be used to obtain new information about known associations between drugs and adverse reactions (Srba et al., 2012). This study retrieved three antineoplastic drugs approved by the US Food and Drug Administration (FDA): Avastin, Berubicin, and Temozolomide.

These three anti-glioma drugs show good efficacy characteristics, but due to the aging of organs in elderly patients, impaired physiological adaptability, and decreased immune function, their tolerance and sensitivity will be reduced, thereby increasing the risk of adverse reactions and complications in elderly patients, and the prognosis is often poor. Clinical doctors must therefore typically adjust treatment plans based on each patient’s unique risk of side effects. We performed a descriptive analysis of spontaneous adverse events recorded in the VigiAccess database in order to compare the variations in adverse reactions associated with these three anti-glioma medications.

## Materials and methods

### Drug samples

Table 1 displays the general information of the three anti-glioma drugs that are available for clinical treatment in our study.

**TABLE 1:** General information of the three anti-glioma drugs studied for clinical treatment.

Drug name and brand name	Structure	Main conditions	First marketing time
Avastin	Recombinant humanized monoclonal antibody	Recurrent, newly diagnosed glioblastomas and other brain tumors	2004
Berubicin	Anticancer drugs of the anthracyclines	Malignant glioma	2021
Temozolomide	Small molecule alkylating agent	Newly diagnosed glioblastoma multiforme, glioblastoma multiforme that recurred or progressed after conventional treatment, or anaplastic astrocytoma	2005

Avastin, an anti-vascular endothelial growth factor (VEGF)-A antibody, was approved by the U.S. Food and Drug Administration (FDA) on 22 February 2008, for the treatment of a range of cancers. Angiogenesis is a hallmark of glioblastoma, and Avastin has been shown through *in vivo* and *in vitro* testing systems to bind to human VEGF and block its biological activity, thereby demonstrating efficacy in clinical trials for glioblastoma. Since its widespread introduction in clinical treatment in 2009, Avastin has shown benefits in both clinical studies and population studies, including a well-documented increase in the overall survival (OS) time of patients with glioblastoma (Zhu et al., 2017). Recently, the FDA granted Berubicin, an anthracycline drug, a fast-track designation for the treatment of patients with glioblastoma multiforme (GBM).

One of the original members of the anthracycline class, doxorubicin, is an O-benzylated derivative of berubicin; the sugar moiety is the only distinction between the two (Cortés-Funes and Coronado, 2007; Martins-Teixeira and Carvalho, 2020). Berubicin is an anthracycline class of anticancer drugs and is one of the most effective chemotherapy drugs currently available. The less-than-ideal drug treatment for GBM is largely attributed to the “blood-brain barrier” structure around the brain, which is designed to prevent toxins and pathogens from entering the brain. However, its existence also prevents many potentially effective drugs from penetrating the brain through the bloodstream or orally. Berubicin is the first anthracycline drug capable of crossing the blood-brain barrier and has a strong killing ability against glioblastoma. Coupled with its wide range of applications as a chemotherapy drug, Berubicin is expected to extend the survival period of patients with glioblastoma.

Temozolomide (TMZ) was approved by the FDA for marketing in the United States on 11 August 1999. As the preferred alkylating agent, it is used as a standard therapy for adult refractory glioblastoma multiforme (GBM) and astrocytoma, easily penetrating the blood-brain barrier (BBB), making it an effective drug for the treatment of gliomas (Lee, 2016). Mismatch repair can identify small nucleotide insertions/deletions and promote cell cycle arrest in the G2/M phase, which leads to the death of GBM cells. It alkylates genomic DNA at the N7 and O6 positions of guanine and the N3 position of adenine, inducing nucleotide mismatches in subsequent replication cycles (Strobel et al., 2019). Nucleotide mismatches lead to the replacement of cytosine with thymine, in contrast to methylated guanine (Lips and Kaina, 2001). This separates TMZ-induced O-6-methylguanine adducts and restores genomic integrity, ensuring the activity of normal cells. Table 1 shows that the structures of the three drugs are not completely the same; except for Berubicin, the other two drugs have been on the market for nearly a decade and have been applied in domestic clinical treatment.

As of July 2024, there are three biosimilars for Avastin and three for Berubicin, while there are currently no biosimilars of Temozolomide on the market. However, in a new study, researchers from Yale University in the United States have developed a TMZ analog for use as a chemotherapy drug for gliomas. The related research results were published in the journal Science on 29 July 2022 (Lin et al., 2022) The study shows that in patients with recurrent or progressive glioblastoma, the original drug and biosimilars of Avastin have similar safety and clinical efficacy (Kumar et al., 2021). Switching between products is only allowed after short-term and long-term studies and clinical use have proven similar efficacy and safety of the original drug and biosimilars.

### Search strategy and data source

A WHO-VigiAccess search was done on 30 July 2024, to find any adverse events that were documented after anti-glioma medication was used clinically. https://www.vigiaccess.org is the webpage where users can log in. Worldwide data on age groups, gender, reporting year, and continents is gathered by WHO-VigiAccess. UMC is able to access medication safety records using the free PIDM database portal, WHO-VigiAccess. VigiAccess is a public resource that provides a statistical representation of the data in VigiBase. The VigiBase database began in 1968 when data were reported by member countries of the International Drug Monitoring Programme (PIDM), initially with the participation of only 10 countries. As of March 2022, it has developed to 155 full member states and 21 associate member states waiting for the approval of full membership. Icsrs reported by member States are mainly reported by health professionals, patients and pharmaceutical companies to the drug regulatory authorities in their countries, which are identified after review and analysis and sent to VigiBase in bulk. The definition is based on the Medical Dictionary for Regulatory Activities (MedDRA) Preferred Terms (PTs) and System Organ Class (SOC). In order to characterize the toxicity spectrum, data for every medication were obtained, and each individual adverse event was recognized using the MedDRA SOC and PT levels that were noted. A number of dictionaries, including the World Health Organization Adverse Reaction Terminology (WHO-ART), are the source of the reporting words used in MedDRA (Sultana et al., 2020). Twenty of the 27 elements in the SOC categorization that were specifically linked to illness symptoms were chosen for examination. We created three severity categories—death, hospitalization, and severe occurrences, including life-threatening events—by grouping the discovered data using outcome codes. The medicine name serves as the search formula, and the active ingredients are screened to provide the desired outcome. To find pertinent studies, we also manually scanned the included studies’ and citations’ reference lists. To arrange and eliminate duplicates from the final search results, Endnote X9 was utilized.

### Disproportionality analysis

Based on disproportionality analysis, we applied two methods for disproportionate reporting: the reporting odds ratio (ROR) (Rothman et al., 2004) and the proportional reporting ratio (PRR) (Evans et al., 2001). The calculation of ROR and PRR is based on the measure of odds imbalance, a method commonly used in AE signal mining (van Puijenbroek et al., 2003). The ROR is calculated to measure the likelihood imbalance of reporting an adverse event (AE) for a specific medication in comparison to other medications. The formula provides the ROR.:

$$ROR=\frac{a\times d}{b\times c}$$

where the numbers in (a), (b), (c), and (d) represent the number of reports for the specific drug and specific AE, the number of reports for the specific drug and other AEs, and the number of reports for other drugs and other AEs. For a given medication and AE combination, a minimum of 5 examples (a ≥5) are needed to guarantee the statistical stability of the ROR computation. An additional metric for measuring the disproportionality of AE reports is the PRR. It is calculated as:

$$PRR=\frac{a\left(c+d\right)}{c\left(a+b\right)}$$

Like ROR, for the particular drug and AE combination (a ≥5) to be deemed legitimate, the PRR computation necessitates a minimum of 5 cases. If both the ROR value and the lower limit of the 95% Confidence Interval (CI) for ROR are larger than 2 (ROR >2) and greater than 1 (Lower limit of 95% CI of ROR >1), then the signal is deemed disproportionate and may be a sign of a safety risk. These criteria ensure that the observed disproportionality is not the result of random variability. By using ROR and PRR in our research, we were able to thoroughly evaluate the disproportionality of ocular issues linked to anti-glioma medication use. The results of the analysis back up pharmacovigilance programs intended to increase drug safety.

### Statistical analysis

This study uses a retrospective quantitative research method, which is a method of studying the past by looking at the results in the present. This research method has fewer conditional restrictions. Excel was used to analyze the gender, age and regional characteristics of the victims of adverse reactions of three anti-glioma drugs from the aspects of current situation, case report, case series analysis and data analysis. The ADR reporting rate for each drug is defined as the number of ADR symptoms for each drug divided by the total number of ADR reports. Common ADRs for each drug refer to the ADR reporting rates of the top 20 symptoms. The incidence of ADR symptoms reported for each drug was calculated and a descriptive comparative analysis was performed. Descriptive variables were categorized using frequency and percentage to derive convincing results.

## Results

### Case description of the study

The WHO-VigiAccess database shows that the earliest adverse reports received for Avastin, Berubicin, and Temozolomide were in 1987, 1985, and 1997, respectively. As of 2024, the World Health Organization has received a total of 80,330 adverse reports for Avastin, 31,533 for Berubicin, and 20,608 for Temozolomide, totaling 132,471. The number of adverse events covered in these adverse reaction reports is 135,782 for Avastin, 46,393 for Berubicin, and 37,533 for Temozolomide. Among the 132,471 reports related to the three anti-glioma drugs shown in Table 2, excluding 6,311 reports with unknown gender, there were 75,515 adverse reaction reports for women and 50,645 for men, with women’s adverse reaction reports significantly more than men, with a male-to-female ratio of 1:1.5, a significant difference. Excluding reports with unknown age, the highest reporting incidence age group is 45–64 years old, mainly the elderly. Most of the reported AEs came from Africa (98.6%). Table 2 also lists basic information.

**TABLE 2:** Characteristics of adverse reactions of three anti-glioma drugs, such as gender and age.

	Avastin	Berubicin	Temozolomide
Number of ADR reports	80,330	31,533	20,608
Female	40,379 (50.3%)	26,443 (83.9%)	8693 (42.2%)
Male	36,734 (45.7%)	4262 (13.5%)	9649 (46.8%)
Unknown	3217 (0.4%)	828 (2.6%)	2266 (11%)
<18	170 (0.2%)	349 (1.1%)	1131 (5.5%)
18–44	5218 (6.5%)	6298 (20%)	2948 (14.3%)
45–64	31,872 (39.7%)	16,954 (53.8%)	6644 (32.2%)
65–74	19,337 (24.1%)	4577 (14.5%)	3022 (14.7%)
>75	10,922 (13.6%)	926 (2.9%)	1113 (5.4%)
Unknown	12,811 (15.9%)	2429 (7.7%)	5750 (27.9%)
Africa	639 (0.8%)	251 (0.8%)	82 (0.4%)
Americas	22,637 (28.2%)	1600 (5.1%)	13,007 (63.1%)
Asia	20,662 (25.7%)	19,223 (61%)	2455 (11.9%)
Europe	31,640 (39.4%)	10,271 (32.6%)	4804 (23.3%)
Oceania	4752 (5.9%)	188 (0.6%)	260 (1.3%)
Before 2010	33,136 (41.2%)	4192 (13.3%)	3735 (18.1%)
2011	5089 (6.3%)	917 (2.9%)	702 (3.4%)
2012	2838 (3.5%)	650 (2.1%)	457 (2.2%)
2013	2601 (3.2%)	1064 (3.4%)	554 (2.7%)
2014	4223 (5.3%)	2527 (8%)	1486 (7.2%)
2015	4203 (5.2%)	1808 (5.7%)	1075 (5.2%)
2016	3833 (4.8%)	1821 (5.8%)	1021 (5%)
2017	3526 (4.4%)	1793 (5.7%)	1315 (6.4%)
2018	4107 (5.1%)	1684 (5.3%)	1585 (7.7%)
2019	5694 (7.1%)	2326 (7.4%)	1914 (9.3%)
2020	2923 (3.6%)	3759 (11.9%)	1425 (6.9%)
2021	2836 (3.5%)	2104 (6.7%)	1447 (7%)
2022	1958 (2.4%)	2223 (7.1%)	1454 (7%)
2023	2020 (2.5%)	2717 (8.6%)	1653 (8%)
2024 (Until July 30)	1343 (1.75)	1948 (6.2%)	785 (3.8%)

### Distribution tables of 20 SOCs for three anti-glioma drugs

The reporting rates for 20 SOCs of the three anti-glioma medications are displayed in Table 3. Avastin-related reporting rates for problems of the musculoskeletal and connective tissue (36.6%), neurological system, and skin and subcutaneous tissue are much greater than those of the other two anti-glioma medications. Temozolomide had a greater reporting incidence for injury, poisoning, and procedural complications than for general disorders and administration site problems (31.9%). Conversely, berubicin has the highest rate of adverse reaction reporting (45.6%) in diseases of the blood and lymphatic system.

**TABLE 3:** Number of adverse reactions and reporting rates for 20 SOCs of three anti-glioma drugs.

System organ class	Avastin(N=80,330)	Berubicin(N=31,533)	Temozolomide(N=20,608)
Blood and lymphatic system disorders	1240(1.5%)	14,368(45.6%)	4625(22.4%)
Cardiac disorders	2890(3.6%)	1508(4.8%)	407(2.0%)
Congenital familial and genetic disorders	157(0.2%)	43(0.1%)	166(0.8%)
Ear and labyrinth disorders	867(1.1%)	138(0.4%)	99(0.5%)
Endocrine disorders	193(0.2%)	81(0.3%)	113(0.5%)
Eye disorders	2384(3.0%)	211(0.7%)	266(1.3%)
Gastrointestinal disorders	12,894(16.1%)	5889(18.7%)	3278(15.9%)
General disorders and administration site conditions	15,685(19.5%)	4720(15%)	6568(31.9%)
Hepatobiliary disorders	3760(4.7%)	1025(3.3%)	715(3.5%)
Immune system disorders	2055(2.6%)	246(0.8%)	236(1.1%)
Infections and infestations	1834(2.3%)	1558 (4.9%)	2134(10.4%)
Injury poisoning and procedural complications	3459(4.3%)	500 (1.6%)	3105(15.1%)
Investigations	10,401(12.9%)	5403 (17.1%)	2481(12%)
Metabolism and nutrition disorders	2580(3.2%)	1022 (3.2%)	1136(5.5%)
Musculoskeletal and connective tissue disorders	29,426(36.6%)	684 (2.2%)	585(2.8%)
Neoplasms benign malignant and unspecified incl cysts and polyps	629(0.8%)	1042 (3.3%)	2043(9.9%)
Nervous system disorders	13,507(16.8%)	1785 (5.7%)	3107(15.1%)
Pregnancy puerperium and perinatal conditions	93(0.1%)	75 (0.2%)	35(0.2%)
Product issues	1126(1.4%)	12 (0.04%)	128(0.6%)
Psychiatric disorders	5344(6.7%)	371 (1.2%)	769(3.7%)
Renal and urinary disorders	3643(4.5%)	349 (1.1%)	493(2.4%)
Reproductive system and breast disorders	1282(1.6%)	138 (0.4%)	58(0.3%)
Respiratory thoracic and mediastinal disorders	4334(5.4%)	1353 (4.3%)	1384(6.7%)
Skin and subcutaneous tissue disorders	13,063(16.3%)	2806 (8.9%)	1658(8%)
Social circumstances	363(0.5%)	27 (0.1%)	89(0.4%)
Surgical and medical procedures	491(0.6%)	71 (0.2%)	890(4.3%)
Vascular disorders	2082(2.6%)	968 (3.1%)	965(4.7%)

The top five types of adverse events (AEs) for anti-glioma drugs are as follows: blood and lymphatic system disorders (20,233 cases, 15.2%), general disorders and administration site conditions (26,973 cases, 20.3%), gastrointestinal disorders (22,061 cases, 16.7%), investigations (18,285 cases, 13.8%), and musculoskeletal and connective tissue disorders (30,695 cases, 23.1%).

### Disproportionality analysis based on blood and lymphatic system disorders

By observing and comparing the SOC distributions of four anti-glioma drugs, it was found that diseases of the blood and lymphatic system were the most common AE. To further compare these four agents, we performed an imbalance analysis according to blood and lymphatic system disorders. ROR and PRR methods were used. According to Table 4, through the disproportionation analysis, we found that the ROR values of the three drugs were: Avastin: 2.15 (3.27–1.41); Berubicin: 67.09 (83.10–54.16); Temozolomide: 37.07 (54.55–25.19). The PRR values of the three drugs were as following: Avastin: 2.15 (3.27–1.41); Berubicin: 65.39 (83.10–54.26); Temozolomide: 36.90 (54.55–25.19). The results indicate that Berubicin seems to be more likely to cause disorders of the blood and lymphatic system than other anti-glioma agents.

**TABLE 4:** Disproportionality analysis based on blood and lymphatic system disorders.

	RoR (95% CI)	PRR (95% CI)
Avastin	2.15 (3.27–1.41)	2.15 (3.27–1.41)
Berubicin	67.09 (83.10–54.16)	65.39 (83.10–54.26)
Temozolomide	37.07 (54.55–25.19)	36.90 (54.55–25.19)

### The most common adverse reactions of three anti-glioma drugs

Table 5 lists the 20 most frequently reported adverse reactions for the three anti-glioma drugs, which are presented as preferred terms within the SOCs. The common adverse reactions of avastin include myalgia, rhabdomyolysis, elevated serum creatine phosphokinase, pruritus, dizziness, etc. Common adverse effects of berubicin include myelosuppression, leukopenia, nausea, vomiting, and neutropenia. The common adverse events of temozolomide were thrombocytopenia, death, nausea, worsening of disease and vomiting. Compared to the other two anti-glioma drugs, Avastin has a significantly higher reporting rate for the adverse reaction of myalgia. Berubicin has the highest reporting rate for adverse reactions related to bone marrow suppression. Among the top 20 adverse reaction reports, some are self-limiting, such as nausea and vomiting, but there are also adverse reactions that require attention, such as neutrophil, white blood cell, and platelet reductions.

**TABLE 5:** Top 20 adverse reactions of anti-glioma drugs.

Avastin (N=80,330)		Berubicin (N=31,533)		Temozolomide (N = 20,608)	
ADR	Report rate %	ADR	Report rate %	ADR	Report rate %
Myalgia	17.83%	Myelosuppression	21.27%	Thrombocytopenia	9.55%
Rhabdomyolysis	6.63%	White blood cell count decreased	12.87%	Death	7.09%
Blood creatine phosphokinase increased	4.83%	Nausea	8.97%	Nausea	7.04%
Pruritus	4.50%	Vomiting	7.99%	Disease progression	6.21%
Dizziness	4.43%	Neutropenia	6.16%	Vomiting	5.19%
Headache	4.02%	Neutrophil count decreased	5.11%	Product use in unapproved indication	4.39%
Rash	4.01%	Leukopenia	4.87%	Fatigue	4.10%
Nausea	3.86%	Pyrexia	4.87%	Off label use	3.97%
Arthralgia	3.79%	Agranulocytosis	4.70%	Neutropenia	3.95%
Muscle spasms	3.51%	Alopecia	4.69%	Seizure	3.65%
Fatigue	3.09%	Febrile neutropenia	3.35%	Pancytopenia	3.63%
Muscular weakness	3.06%	Thrombocytopenia	3.00%	Drug ineffective	3.41%
Asthenia	2.94%	Anaemia	2.78%	Platelet count decreased	3.20%
Diarrhoea	2.68%	Diarrhoea	2.72%	Pyrexia	2.69%
Pain in extremity	2.58%	Asthenia	2.67%	Asthenia	2.57%
Pain	2.33%	Granulocytopenia	2.20%	Leukopenia	2.47%
Abdominal pain	2.14%	Decreased appetite	1.82%	Malignant neoplasm progression	2.44%
Dyspnoea	2.02%	Hepatic function abnormal	1.67%	Diarrhoea	2.35%
Myopathy	1.92%	Constipation	1.51%	Myelosuppression	2.30%
Drug interaction	1.79%	Dyspnoea	1.37%	Rash	2.25%

### Serious adverse events of three anti-glioma drugs

**FIGURE 1:**
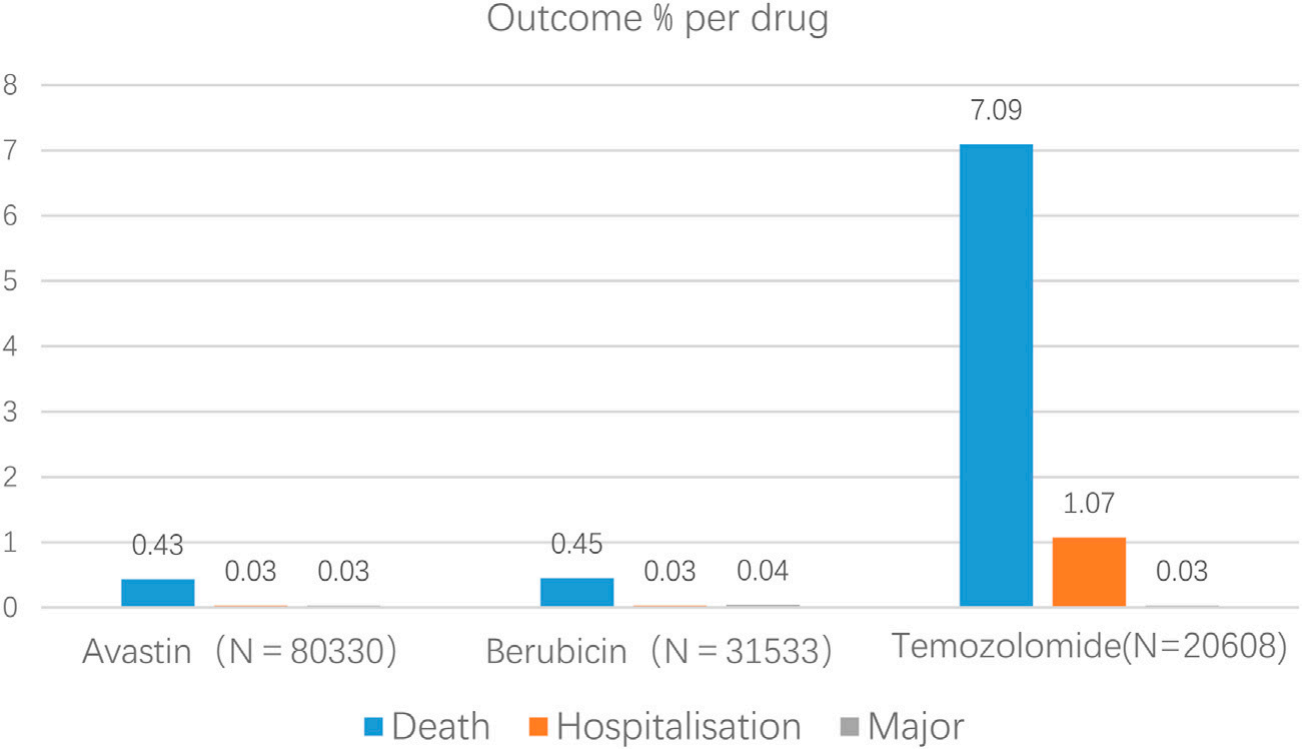
Results of antiglioma medication-related occurrences at the preferred term level (primary events comprised of congenital abnormalities, impairments, and life-threatening events).

By using the database, we can also determine the primary side effects connected to anti-glioma medications, such as fatalities, hospital stays, and life-threatening incidents. Temozolomide, Berubicin, and Avastin have respective rates of 8.19%, 0.52%, and 0.49% of major adverse events (Figure 1).

### Commonalities and differences in the most common adverse reactions of three anti-glioma drugs

By comparing the top 20 adverse reactions reported in the SOCs for each anti-glioma drug, we found that there are 81 common adverse reactions among the three drugs at the PTs level. Table 6 lists all the commonalities. The most common adverse reactions are gastrointestinal disorders, with the top five reported being dysphagia, gastrointestinal hemorrhage, abdominal discomfort, constipation, and abdominal pain; followed by general disorders and administration site conditions, with the top five reported being lack of drug effectiveness, fever, death, pain, and malaise.

**TABLE 6:** Common adverse reactions of three anti-glioma drugs.

System organ classes	ADRs	Signal N
Blood and lymphatic system disorders	Anaemia, Thrombocytopenia	2
Cardiac disorders	Atrial fibrillation, Tachycardia, Myocardial infarction, Arrhythmia	4
Ear and labyrinth disorders	Vertigo	1
Gastrointestinal disorders	Dysphagia, Gastrointestinal haemorrhage, Abdominal discomfort, Constipation, Abdominal pain, Vomiting, Nausea, Dyspepsia, Abdominal distension, Abdominal pain upper, Diarrhoea, Gastrointestinal disorder	12
General disorders and administration site conditions	Drug ineffective, Pyrexia, Death, Pain, Malaise, Fatigue, Condition aggravated, Oedema peripheral, Asthenia, Oedema, Chest pain	11
Hepatobiliary disorders	Liver injury, Hepatic function abnormal	2
Immune system disorders	Hypersensitivity	1
Infections and infestations	Sepsis, Pneumonia, Urinary tract infection	3
Injury, poisoning and procedural complications	Toxicity to various agents, Off label use, Fall, Product use in unapproved indication	4
Investigations	Transaminases increased, Aspartate aminotransferase increased, Hepatic enzyme increased, Alanine aminotransferase increased, Weight decreased, Gamma-glutamyltransferase increased	6
Metabolism and nutrition disorders	Decreased appetite, Hypokalaemia, Dehydration, Hyponatraemia, Hyperglycaemia	5
Musculoskeletal and connective tissue disorders	Bone pain, Pain in extremity, Back pain, Arthralgia, Muscular weakness, Myalgia	6
Nervous system disorders	Syncope, Hypoaesthesia, Headache, Dizziness, Neuropathy peripheral, Tremor	6
Psychiatric disorders	Insomnia, Anxiety, Depression, Confusional state	4
Renal and urinary disorders	Renal failure, Acute kidney injury	2
Respiratory, thoracic and mediastinal disorders	Dyspnoea, Respiratory failure, Interstitial lung disease, Cough	4
Skin and subcutaneous tissue disorders	Erythema, Rash, Pruritus, Alopecia, Urticaria, Rash erythematous	6
Vascular disorders	Hypertension, Hypotension	2

A comparison of the three medications' top particular side effects is shown in Table 7. When comparing the top 20 adverse reactions in the SOCs for each anti-glioma drug, the PTs for gastrointestinal disorders, general disorders and administration site conditions, infections, injuries, poisoning and procedural complications, investigations, metabolic and nutritional disorders, nervous system disorders, psychiatric disorders, skin and subcutaneous tissue disorders, and vascular disorders are all different among the drugs. In terms of gastrointestinal disorders, the number of unique symptoms for the three drugs is 4, 2, and 2, respectively. For infections, the number of unique symptoms is 1, 3, and 12, respectively.

**TABLE 7:** Differences in adverse reactions of three anti-glioma drugs.

System organ classes	Avastin	Berubicin	Temozolomide
Blood and lymphatic system disorders		Erythropenia, Febrile bone marrow aplasia, Leukocytosis, Bicytopenia	Aplastic anaemia, Cytopenia, Immune thrombocytopenia, Bone marrow failure
Cardiac disorders	Bradycardia, Coronary artery disease, Angina pectoris	Cardiotoxicity, Left ventricular dysfunction, Cardiac flutter, Dilated cardiomyopathy, Cardiomyopathy, Cardiac dysfunction	
Congenital, familial and genetic disorders			Hypermutation
Ear and labyrinth disorders	Tinnitus		
Endocrine disorders		Hypothyroidism	Diabetes insipidus
Eye disorders	Eye pain, Periorbital oedema, Cataract, Eye swelling, Eyelid oedema, Diplopia	Lacrimation increased	
Gastrointestinal disorders	Swollen tongue, Flatulence,Lip swelling, Pancreatitis acute	Aphthous ulcer, Mouth ulceration	Large intestine perforation, Rectal haemorrhage
General disorders and administration site conditions	Gait inability, Feeling abnormal, Unevaluable event, Drug interaction, Face oedema, Drug intolerance, Peripheral swelling	Extravasation, Injection site reaction, Administration site extravasation, Mucosal inflammation, Hyperpyrexia	Adverse event, Therapy non-responder, No adverse event, Disease recurrence, Treatment failure
Hepatobiliary disorders	Hepatic steatosis, Hepatitis cholestatic		Acute hepatic failure, Cholestasis, Hepatic cytolysis
Immune system disorders		Anaphylactic shock, Anaphylactic reaction	
Infections and infestations	COVID-19	Cystitis, Abscess, Bronchitis	Staphylococcal infection, Herpes simplex encephalitis, Pneumocystis jirovecii infection, *Candida* infection, Meningitis, Pneumonia aspiration, Diverticulitis, Oral candidiasis, Wound infection, Pneumocystis jirovecii pneumonia, Aspergillus infection, Nasopharyngitis
Injury, poisoning and procedural complications	Product administration error, Intentional overdose, Muscle injury, Contraindicated product administered, Product prescribing error, Tendon rupture	Maternal exposure during pregnancy, Infusion related reaction	Product dispensing error, Product administered to patient of inappropriate age, Inappropriate schedule of product administration, Incorrect dose administered, Accidental overdose, Subdural haematoma, Radiation necrosis, Product use issue, Head injury
Investigations	Red blood cell sedimentation rate increased, Blood triglycerides increased, Blood creatine phosphokinase increased, Blood pressure increased, Weight increased, Heart rate increased, Blood cholesterol increased, International normalised ratio increased	Blood pressure decreased, Ejection fraction decreased	C-reactive protein increased, Red blood cell count decreased, Lymphocyte count decreased, Haematocrit decreased
Metabolism and nutrition disorders	Hyperlipidaemia, Diabetes mellitus, Hyperkalaemia, Hypercholesterolaemia, Gout, Hypoglycaemia	Appetite disorder	Hypocalcaemia, Hypoalbuminaemia, Hypophagia
Musculoskeletal and connective tissue disorders	Musculoskeletal stiffness, Rhabdomyolysis, Muscle atrophy, Myositis, Neck pain, Arthritis, Muscle spasms, Arthropathy, Joint swelling, Musculoskeletal pain, Muscle disorder, Tendonitis, Mobility decreased, Myopathy		
Neoplasms benign, malignant and unspecified (incl cysts and polyps)		Breast cancer, Second primary malignancy, Acute leukaemia, Breast cancer metastatic, Myeloid leukaemia, Metastases to bone, Metastases to lymph nodes	Malignant melanoma, Neoplasm malignant, Tumour necrosis, Brain neoplasm malignant, Metastatic malignant melanoma, Glioblastoma, Astrocytoma malignant, Glioblastoma multiforme, Recurrent cancer, Brain neoplasm, Tumour pseudoprogression, Neoplasm, Metastases to central nervous system, Neoplasm recurrence
Nervous system disorders	Loss of consciousness, Movement disorder, Amnesia, Balance disorder, Disturbance in attention	Polyneuropathy, Taste disorder	Generalised tonic-clonic seizure, Epilepsy, Brain oedema, Aphasia, Speech disorder, Hydrocephalus, Hemiparesis, Cerebral haemorrhage
Psychiatric disorders	Nervousness, Aggression, Suicidal ideation, Nightmare, Suicide attempt, Completed suicide, Irritability, Abnormal dreams, Sleep disorder, Libido decreased, Hallucination, Depressed mood	Restlessness	Disorientation, Mental status changes
Renal and urinary disorders	Azotaemia, Renal disorder, Pollakiuria, Nocturia, Chromaturia		Proteinuria, Urinary incontinence
Reproductive system and breast disorders	Gynaecomastia, Erectile dysfunction	Amenorrhoea	
Respiratory, thoracic and mediastinal disorders	Dysphonia, Throat irritation		Hypoxia, Respiratory distress, Atelectasis, Acute respiratory distress syndrome, Lung disorder, Pulmonary thrombosis, Lung infiltration
Skin and subcutaneous tissue disorders	Eczema, Angioedema, Rash papular, Psoriasis, Rash macular, Photosensitivity reaction, Blister, Skin exfoliation	Nail discolouration, Skin necrosis, Skin hyperpigmentation	Petechiae, Toxic epidermal necrolysis, Drug eruption, Stevens-johnson syndrome, Drug reaction with eosinophilia and systemic symptoms
Social circumstances	Loss of personal independence in daily activities		
Surgical and medical procedures			Therapy change, Surgery, Brain operation, Hospitalisation, Therapy cessation, Hospice care, Therapy interrupted
Vascular disorders	Vasculitis	Circulatory collapse, Thrombophlebitis, Phlebitis	Embolism

## Discussion

Owing to the intrinsic constraints of clinical trials, including rigorous inclusion criteria, small sample sizes, and short follow-up periods, the Spontaneous Reporting System (SRS) has been employed in pharmacovigilance to evaluate the safety of suspected adverse events. being crucial to the identification of signals. The majority of research on drug safety signals now comes from three main databases: WHO-VigiBase^®^, FDA Adverse Event Reporting System (FAERS), and EudraVigilance Data Analysis System (EVDAS). With the launch of WHO-VigiAccess in 2015, the World Health Organization hopes to make information from VigiBase^®^, its global database of possible drug adverse effects, available to the general population. Some known clinical linkages and previously undiscovered medication AE associations will be revealed by data mining of the WHO-VigiAccess database.

Data from WHO-VigiAccess show that 35.3% of the adverse event reports related to the three anti-glioma drugs came from Europe, followed by Asia. A survey study on brain cancer in New Zealand showed that from 1995 to 2020, over a 25-year period, there were 6,677 glioma cases, with the age-standardized rate (World Health Organization world standard) being 604 per 100,000 for males and 395 for females (Elwood et al., 2022) In the United States, approximately 18,500 people are diagnosed with malignant gliomas each year (Raizer et al., 2015), and a higher number of adverse event reports regarding anti-glioma drugs are observed. Before the approval of effective chemotherapy drugs for malignant gliomas in the United States, the estimated direct medical cost for surgery and radiation therapy per patient close to a hundred thousand dollars The addition of temozolomide (TMZ) and Avastin to the glioma treatment regimen increased the overall cost of glioma treatment. Although current medical expenses reduce the initial burden, these costs increase with the occurrence of the disease (Raizer et al., 2015), not to mention in developing countries. All things considered, there are a lot of intricate elements that could account for Africa’s low rates of spontaneous reporting. Several authors have identified significant obstacles to the development of health systems in Africa, such as inadequate national health infrastructure and systems, a lack of awareness, a lack of information in official health professional training curricula, and a lack of enthusiasm on the part of health professionals (Ampadu et al., 2016; Sabblah et al., 2014; Rouamba et al., 2020).

Adverse reaction report data show that the number of females is much greater than that of males, and the adverse events of anti-glioma drugs in the age group of 45–64 are the most, followed by the age group of 65–74. As age increases, physiological functions gradually decline, and the elderly often have various comorbidities, which affect the metabolic process of drugs in the body and greatly increase the risk of adverse events. A review of the Japanese drug adverse event report database revealed a significant correlation between the use of Avastin and the incidence of central nervous system ischemia after Avastin administration (adjusted reporting odds ratio (ROR): 2.68, 95% confidence interval (CI): 2.00–3.59, *p* < 0.001). Moreover, there was a substantial correlation (*p* < 0.001) between the use of Avastin and the diagnosis of glioma. Logistic regression indicated that central nervous system ischemia caused by Avastin is related to glioma, potential hypertension, and aging. It has been reported that poor prognosis mostly occurs in elderly patients (over 60 years old) (Sugimoto et al., 2022) Therefore, although adverse events occur in all age groups, the highest incidence rate is in the 45–64 age group.

AEs with a reporting rate >1% are generally considered the most common (Chen et al., 2019). Adverse events of the three anti-glioma drugs, including death, hospitalization, and life-threatening events, are not common, but the death event of temozolomide is 7.09%, much more than Avastin and Berubicin. The most common adverse reactions of the three anti-glioma drugs are blood and lymphatic system diseases. Through the VigiAccess database, we found that the most common AEs of the three drugs are bone marrow suppression, myalgia, leukopenia, thrombocytopenia, nausea, vomiting, death, rhabdomyolysis, disease progression, and neutrophil decrease.

Using Avastin as an example, researchers looked up 20 publications on the drug’s side effects and discovered that the majority of them were related to hematological system and cardiovascular disorders. Research has indicated that there are five typical side effects associated with Avastin use: proteinuria, thrombotic events, bleeding, cerebral hemorrhage, and hypertension. Proteinuria, hypertension, hemorrhage, fever, and convulsions were the most frequent side events, according to a phase II trial conducted in Japan (Friedman et al., 2009) Although the incidence of bleeding events is relatively high, at 13.4%, it is mainly grades 1 to 2 ADRs. Avastin-related bleeding can be divided into tumor-related bleeding (especially hemoptysis during the treatment of non-small cell lung cancer) and skin mucosal-related bleeding, with the latter being more common, and the most common being epistaxis, which can generally be relieved without medical intervention. 64 glioblastoma patients receiving Avastin anticoagulant therapy and 64 glioma patients not receiving anticoagulant therapy have been analyzed by certain researchers. The frequency of severe cerebral hemorrhage was found to be within an acceptable range, but the incidence of other bleeding and intracranial hemorrhage was found to be considerably greater in individuals treated with anticoagulants than in patients treated with Avastin alone (Nagane et al., 2012). Thus, when taking Avastin in clinical practice, it's important to keep a watchful eye out for any adverse medication responses, keep an eye on indicators like blood pressure and coagulation function, and treat with symptoms as soon as they arise.

The U.S. Food and Drug Administration’s Adverse Event Reporting System (FAERS) database checked the adverse events (AEs) of Avastin, especially bleeding adverse events. According to the data, 892 key preferred words (PWs) were spread across 25 organ systems in 96,477 cases of adverse events linked to Avastin that were reported between January 2004 and September 2022 using the proportionate reporting ratio (PRR) and reporting odds ratio (ROR). General disorders, administration site problems, blood and lymphatic system diseases, poisoning, and procedural complications are the main areas of concern for the System Organ Class (SOC). Avastin-related bleeding was documented in 2,847 cases in total, and 37 bleeding signals were discovered. In vascular, gastrointestinal, and nervous system disorders, bleeding signals were mostly localized at the SOC level, and additional examination of the clinical data pertaining to bleeding signals was carried out.

The databases WHO-VigiAccess and FAERS, which are used to evaluate post-marketing drug vigilance, demonstrate variations in the kinds and frequency of adverse reactions related to the blood system that are brought on by anti-glioma drugs. Because adverse events are reported voluntarily, information on reported events may be lacking in the FAERS and WHO-VigiAccess databases, which do not provide a complete and comprehensive statistical representation of adverse events. Conversely, the FAERS database, with the help of experts and sophisticated analysis software, can, in the majority of situations, display individual reports for each adverse reaction and more precisely select case reports that satisfy the requirements (Mikami et al., 2021). In order to prevent giving the wrong advice, WHO-VigiAccess may need to give the public access to more report information in order to filter possible connections between medications and adverse reactions.

Another significant adverse event of anti-glioma drugs is the increased probability of gastrointestinal dysfunction. Taking temozolomide as an example, its most common toxicities are gastrointestinal issues, such as nausea, vomiting, and anorexia. A retrospective study on the toxicity profile of 300 Korean patients with malignant gliomas treated with temozolomide indicates that 209 patients (69.7%) reported 618 types of toxicities. Non-hematological toxicities were more common than hematological ones. There were 133 cases of nausea (44.3%), 111 cases of vomiting (37.0%), 43 cases of anorexia (14.3%), 32 cases of headache (10.7%), 31 cases of fatigue (10.3%), and 31 cases of alopecia (10.3%) (Bae et al., 2014) Although temozolomide exhibits various toxicities, 84.8% of the toxicities are grade 1 or 2, which patients may tolerate. On the other hand, gastrointestinal problems like nausea (44.3%) and vomiting (37.0%) are among the most frequent side effects of temozolomide. Nausea and vomiting are among the most frequent, upsetting, and potentially dangerous side effects of chemotherapy, negatively impacting cancer patients’ physical and mental wellbeing (Hawkins and Grunberg, 2009). As a result, medical professionals and nurses may need to actively monitor gastrointestinal toxicity in addition to giving preventative antiemetics (Middleton and Lennan, 2011). An EORTC 1608 STEAM trial showed that compared to radiotherapy alone (Group A), temozolomide alone at 150 mg (Group B) exhibited two dose-limiting toxicities: the main toxicities included gastrointestinal disease, hepatotoxicity, and neutropenia. Our results show that the 10 most common AEs manifest as bone marrow suppression, myalgia, leukopenia, thrombocytopenia, nausea, vomiting, death, rhabdomyolysis, disease progression, and neutrophil decrease, including cancer-related adverse reactions. According to the definition of AEs, the occurrence of these adverse reactions is related to the timing of drug administration, and they may reoccur or worsen upon re-exposure to the drug.

Research suggests that the dosage of avastin affects how severe its adverse effects are. Higher Avastin dosages may have an impact on survival benefits (Lorgis et al., 2012). Furthermore, greater anti-VEGF treatment doses may cause more hypoxia in animal models, which may make tumors more aggressive (Keunen et al., 2011). Hypoxia can also result from long-term usage of anti-VEGF/VEGFR at high doses, as reported by Tamura et al. (2019). According to Weathers and de Groot (2014), hypoxia made worse by anti-angiogenic therapy may be the root of a sequence of events in cancers when there has been extensive vascular pruning.

Whether the combination therapy of Avastin and temozolomide can be applied in the clinic also requires further research. A study on the combination of the two drugs with radiotherapy for newly diagnosed glioblastoma suggests that compared to the placebo group, the incidence of serious adverse events and adverse events of grade 3 or higher in the Avastin group are usually related to Avastin, and the incidence of arterial thrombotic events in the Avastin group has increased. The combination of Avastin with standard radiotherapy-temozolomide for the treatment of newly diagnosed glioblastoma did not improve overall survival, but increased the median progression-free survival by 4.4 months while maintaining quality of life and functional status: however, adverse events related to Avastin treatment have increased (Chinot et al., 2014) Therefore, further research is needed to determine whether combination therapy in the clinic is feasible, and the dosage and impact of Avastin to ensure the selection of more suitable treatment plans for patients.

The risk of cerebrovascular and cardiovascular diseases, as well as how different racial/ethnic/ancestry groups respond to pharmacological therapeutic therapies for these ailments, vary significantly (Centers for Disease Control and Prevention, 2001; Thomas et al., 2010; Shaw et al., 2008; McDowell et al., 2006). For instance, those with African ancestry are more likely than those with European American ancestry (EA) to suffer cardiovascular disease (CVD) (Centers for Disease Control and Prevention, 2001). It is not just thrombotic disease that has been observed to have a varied racial or ethnic risk for illness or treatment failure. For instance, among all ethnic groups worldwide, those of Asian heritage are more likely to get intracerebral hemorrhage (ICH). Additionally, Americans are more likely to have AA than people with European descent (An et al., 2017).

In particular, it is debatable whether race and ethnicity should be taken into account when making clinical decisions and in US FDA package inserts because these factors do not fairly represent populations within subgroups and are muddled by social determinants of health and health disparities. These two words are frequently employed as proxy genetic markers of therapy response and illness risk. As demonstrated, various clinical (comorbidities) and environmental (drug interactions, tobacco use) factors frequently account for the apparent correlation between bad medication outcomes and race. Along with other characteristics frequently considered when making treatment decisions (such as liver/kidney function and BMI), race and ethnicity may be important variables that can be used to predict ADR in some circumstances where pharmacogene variability is spatially different.

The use of spontaneous reporting system databases has some important implicit limitations because reports can be affected by notoriety bias, selection bias, and under-reporting (Faillie, 2019) As observed in the current study results that reported some AEs, the missing data cannot be attributed to either males or females or age groups. In addition, since the World Health Organization’s VigiAccess database is cumulative data, the annual ADR cannot be obtained. Further data mining will therefore not be possible. This study compared the ADR reporting rates of several medications by gathering the number of PTs and ADRs over time.

## Conclusion

Anti-glioma drugs are an essential part of immunotherapy. The study shows that WHO-VigiAccess reported 132,471 adverse reactions caused by anti-glioma drug treatment. The adverse reactions of these drugs mainly focus on diseases of the blood and lymphatic system, general diseases and administration site conditions, and gastrointestinal disorders. It reconfirms the adverse reaction symptoms of diseases of the blood and lymphatic system and gastrointestinal disorders. In addition, the musculoskeletal and connective tissue diseases caused by Avastin and the nervous system disorders caused by temozolomide are also very prominent. Although most ADRs that occur during the treatment process are mild, grades 1 to 2, attention should still be paid to common ADRs in clinical applications, be vigilant for the occurrence of severe ADRs, and terminate treatment in time if necessary to avoid fatal ADRs.

It is recommended that nations do proactive safety research on biological agents in order to ascertain the causal association between adverse reactions and pharmaceuticals. Additionally, these study findings can be made available to the general public in open-access databases to help them better understand the side effects of biotechnology medications.

## Data Availability

The original contributions presented in the study are included in the article/Supplementary Material, further inquiries can be directed to the corresponding authors.
